# Bicuspid Aortic Valve Disease-Associated Aortopathy in Pediatric Subjects—From Traditional Assessment to Current Advances and Future Perspectives: A Narrative Review

**DOI:** 10.3390/medsci14050567

**Published:** 2026-09-13

**Authors:** Oana Iulia Man, Lucia Agoston-Coldea, Cecilia Lazea

**Affiliations:** 1 Department of Internal Medicine, Iuliu Hațieganu University of Medicine and Pharmacy, 400012 Cluj-Napoca, Romania; oana.man92@gmail.com (O.I.M.); luciacoldea@yahoo.com (L.A.-C.); 2 2nd Department of Internal Medicine Clinic, Emergency County Hospital, 400006 Cluj-Napoca, Romania; 3 Department 8, Mother and Child, Iuliu Hațieganu University of Medicine and Pharmacy, 400370 Cluj-Napoca, Romania; 4 1st Pediatrics Clinic, Emergency Pediatric Clinical Hospital, 400370 Cluj-Napoca, Romania

**Keywords:** bicuspid aortic valve disease, pediatric aortopathy, circulating biomarkers, multimodal imaging

## Abstract

**Background**: Bicuspid aortic valve disease (BAVD) is the most frequent congenital heart disease, occurring either as an isolated lesion or in association with other congenital cardiovascular malformations, with variable patterns of progression and risk of valvular and vascular complications. Studies addressing pediatric bicuspid aortopathy are still in their infancy. This narrative review aims to provide a comprehensive perspective on the current scientific evidence regarding BAVD-associated aortopathy in pediatric patients, underscoring multiple challenges in initial diagnosis, long-term surveillance, and therapeutic decision-making, and focusing on the potential roles of circulating biomarkers and advanced multimodal imaging tools that may improve individualized risk stratification. **Methods**: Despite the narrative design of this review, a structured search of the current available literature was performed to identify studies addressing pediatric BAV, associated aortopathy, biomarkers, vascular remodeling, and multimodal imaging. Priority was given to pediatric cohorts, longitudinal studies, consensus documents, and contemporary guidelines. The search was conducted in the online databases PubMed/Medline and Web of Science for English-language original articles published in the last 10 years, up to May 2026. We used the following main terms: “Bicuspid Aortic Valve Disease” [MeSH], “Aorta” [MeSH], “Infant” [MeSH], “Child” [MeSH], “Adolescent” [MeSH], combined by Boolean operators with secondary keywords: “pathogenesis”, “mechanism”, “progression”, “multimodal imaging”, “echocardiography”, “cardiovascular magnetic resonance imaging”, “computed tomography”, “circulating biomarkers”. The retrieved studies were screened for eligibility using previously established inclusion and exclusion criteria. A total of 63 studies were included in the analysis for this narrative review. **Results**: Two main theories underpin the etiopathogenesis of aortopathy associated with the bicuspid aortic valve, positing that genetic factors predispose the aortic wall to remodeling in an abnormal hemodynamic environment. Embryological development and dysregulation of molecular and cellular structures are also intertwined during the formation and progression of the aortic valve with two semilunar cusps, resulting in consequent alterations in the aortic wall’s architectural organization. Advances in molecular studies have highlighted circulating biomarkers with potential utility in predicting aortopathy, as they are involved in extracellular matrix remodeling, endothelial dysfunction, and aortic valve calcification, including matrix metalloproteinases (MMPs) and their tissue inhibitors (TIMPs), transforming growth factor-β (TGF-β), and microRNAs. In addition, multimodal imaging techniques have emerged as essential tools for assessing the morphology and function of cardiovascular structures, particularly aortic biomechanical properties and hemodynamic abnormalities, at the time of initial diagnosis and during regular monitoring. Taken together, blood biomarkers and imaging parameters of aortic remodeling and flow disturbances might gain increasing prognostic value in pediatric BAVD-associated aortopathy. However, larger longitudinal studies are required for clinical validation beyond research settings. Although guidelines on the management of BAVD are available for adults, they are not entirely applicable to children, who are undergoing continuous somatic growth that affects diagnostic possibilities and therapeutic options. **Conclusions**: Despite growing literature in the realm of BAVD and related conditions, the management of pediatric patients remains challenging in daily clinical practice, as adult guidelines cannot be completely applied to children. Given the heterogeneity and complexity of pathogenic mechanisms, clinical presentations, natural history, and outcomes, future research is warranted to explore the progression profiles of valvular and vascular disorders associated with BAV in children and to achieve an optimal approach to pediatric bicuspid aortopathy.

## 1. Introduction

More than 500 years ago, the aortic valve with two semilunar cusps was first described by Leonardo da Vinci, and later by Drs. William Osler and Maude Abbot. Despite the growing literature addressing this valvulo-aortopathy, it still creates significant challenges for echocardiographers, cardiologists, and cardiac surgeons [1,2].

Bicuspid aortic valve disease (BAVD) is the most common congenital heart disease, with a global prevalence of 0.5–2%, affecting predominantly men, with a male-to-female ratio of 3:1, and a paramount propensity for valvular and vascular complications, such as aortic valve stenosis or regurgitation, aortic aneurysm, and eventually acute aortic syndromes through dissection or rupture, which may become life-threatening conditions [2,3]. A bicuspid aortic valve can occur either as an isolated cardiac defect or associated with other congenital heart diseases, with variable patterns of progression and aortic dilatation [4].

Dilatation of the aorta is a primary “aortopathy”, commonly associated with this aortic valve structural malformation, and it is defined based on the value of the Z-score ≥ 2.1 in pediatric patients, while an increase of 1.1–1.5 times above the normal absolute size of the aorta is the diagnostic criterion applied in adults [5].

Among the causes of pediatric aortopathies, BAV is the most frequent, along with connective tissue disorders, and it mainly affects the thoracic aorta; however, specific management guidelines remain limited, leading to variations in clinical practice [6]. Most complications occur in adulthood, but early-onset disease may also occur in children and adolescents, requiring systematic checks and close monitoring through imaging, genetic testing, and family screening [7]. Together with sports restrictions depending on the severity of aortic valve regurgitation, stenosis, or aortic enlargement, medical therapy options and surgical interventions may be required in 12–15% of affected children and adolescents, including percutaneous and surgical procedures [8].

Therefore, due to the high prevalence in the general population and the associated lifelong increased risk for adverse valvular and vascular events, BAVD places a considerable burden on public health, requiring close monitoring. As for etiopathogenesis, in BAVD, aortopathy reflects interconnected embryological factors, as well as genetic, epigenetic, cellular, molecular, and flow-mediated mechanisms, rather than a single cause. A major concern in current research is integrating these mechanisms and developing early blood and imaging biomarkers to better predict the risk of developing complications, even from childhood. As adult-focused guidelines cannot be directly applied to the pediatric population, which differs primarily in somatic growth, risk profiles, and developmental aspects, optimal management of these patients requires genetic testing, cardiovascular imaging, medical therapy, lifestyle modifications, and surgical guidance adapted to growing children [6].

Objective: This paper bridges key knowledge gaps in the literature by offering a holistic framework for understanding the intertwined mechanistic pathways driving bicuspid aortic valve disease and associated conditions in infants, children, and adolescents. Given the heterogeneity in nomenclature, study designs, included patient characteristics, and data complexity across the current literature, this review adopts a narrative approach to summarize various findings. The aim of this narrative review is to summarize current evidence regarding the mechanisms, biomarkers, imaging characteristics, and clinical determinants associated with the development and progression of BAV-related aortopathy in pediatric populations, with particular emphasis on factors that may improve individualized risk stratification. We hypothesize that this integrative perspective will address diverse components of the valvulo-aortopathy complex, highlighting multiple challenges in initial diagnosis, long-term surveillance, and therapeutic decision-making, as well as future research trends in pediatric subjects.

## 2. Materials and Methods

### 2.1. Search Strategy

Although this article was intended as a narrative review, a structured literature search was performed to find relevant studies on pediatric BAV, associated aortopathy, biomarkers, vascular remodeling, and multimodal imaging. We systematically searched the online databases PubMed/Medline and Web of Science for English-language original articles published within the last 10 years, up to May 2026, following established guidelines for narrative reviews, as this format was deliberately chosen over a systematic or scoping review. The search was conducted using the following Medical Subject Headings (MeSH) terms: “Bicuspid Aortic Valve Disease” [MeSH], “Aorta” [MeSH], “Infant” [MeSH], “Child” [MeSH], “Adolescent” [MeSH]. We also used secondary keywords related to the topic to capture additional sources addressing the objective of the review, as follows: “pathogenesis”, “mechanism”, “progression”, “multimodal imaging”, “echocardiography”, “cardiovascular magnetic resonance imaging”, “computed tomography”, “circulating biomarkers”. Boolean operators (AND, OR) were used to combine search terms, ensuring pertinent and precise results. Duplicates were removed based on DOI. Article titles, including those retrieved from the reference lists of previous studies, were screened for eligibility. After reading the abstracts, we revised and manually selected the papers for our qualitative analysis, based on the inclusion and exclusion criteria described below. Priority was given to pediatric cohorts, longitudinal studies, consensus documents, and contemporary guidelines.

### 2.2. Inclusion and Exclusion Criteria

We included: (1) original research articles published in peer-reviewed journals, prospective and retrospective observational studies, experimental studies, and clinical trials investigating aortopathy associated with bicuspid aortic valve disease in the pediatric population; (2) studies on humans, focusing on bicuspid aortopathy in young subjects, especially aged < 18 years old; (3) studies published in English; (4) studies providing a rigorous methodology protocol.

Studies were excluded if they met the following criteria: (1) were not peer-reviewed or were non-original articles, such as case reports, editorials, letters to the editor, conference abstracts, or duplicates; (2) contained unpublished data; (3) were studies on animals or on an older population; (4) focused on unrelated topics or did not address the objective of our work, as previously mentioned.

**Figure 1:**
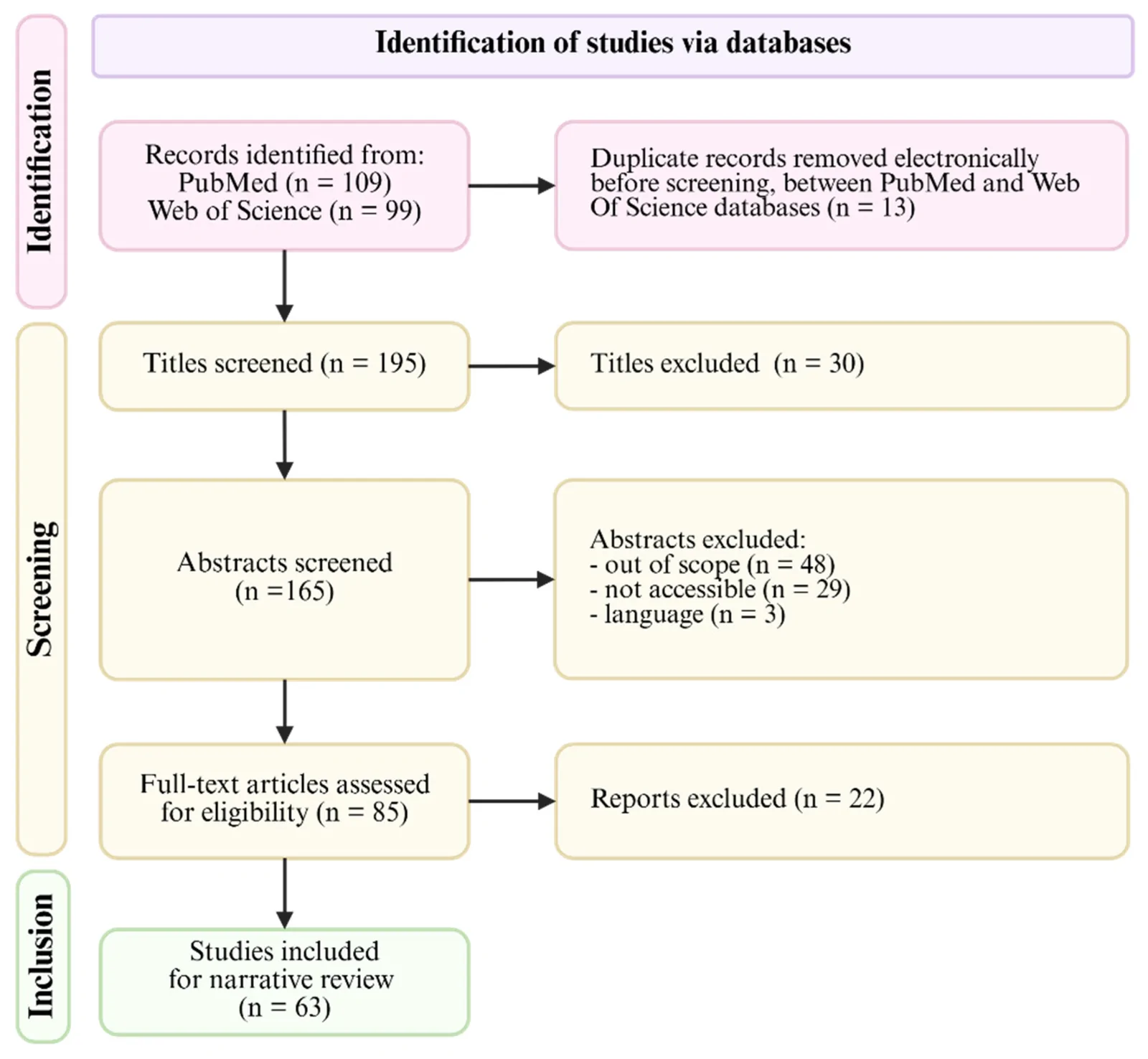
PRISMA flow diagram of the study selection process. Created in BioRender. Man, O. (2026) https://BioRender.com/knnqnys (accessed on 29 August 2026).

The PRISMA flow diagram (Figure 1) was designed to illustrate the search methodology. After the initial search using the main and previously mentioned secondary terms, 208 studies were identified. After removing duplicates, 195 articles were screened by title, and 30 were excluded. Thus, 165 articles remained and were screened based on the abstracts, and 80 were excluded because they were out of scope (n = 48), not accessible (n = 29), or not written in English (n = 3). Therefore, 85 full-text articles were assessed for eligibility, and 63 original studies were included in the narrative review.

### 2.3. Data Extraction and Synthesis

Data were extracted on bicuspid aortic valve-associated aortopathy in patients <18 years old, with particular emphasis on the potential utility of circulating biomarkers and advanced imaging parameters. The main findings of the included papers were summarised narratively. Epidemiological data, nosological aspects, mechanistic insights, and natural history were also briefly discussed to provide a more comprehensive perspective on the topic.

No statistical analyses were conducted, as this review employs a qualitative synthesis approach to highlight the challenges of managing aortic complications associated with the aortic valve phenotype in pediatric subjects, focusing on factors that may improve individual risk stratification.

## 3. Results

### 3.1. Nomenclature and Classification of Bicuspid Aortic Valve Disease and Associated Aortopathy

The congenital BAV is considered a valvulo-aortopathy, characterised by a heterogeneous spectrum of phenotypes, associated conditions, evolution, progression, and potential complications [9]. The term aortopathy refers to a large spectrum of disorders predisposing to dilation, aneurysm, dissection, or rupture of the aorta. In the pediatric population, the most affected segment is the thoracic aorta, with the highest prevalence in bicuspid aortic valve disease and connective tissue disorders [6].

In 2007, Sievers and Schmidtke defined congenital BAV as a group of conditions characterized by deformed aortic valves with two raphes and two functional cusps. They proposed a classification system based on three characteristics: the number of raphes, the spatial position of cusps and raphes, and the functional status of the valve. Thus, BAV was classified into three main types: 0 (no raphe), 1 (one raphe), and 2 (two raphes). The raphe positioning was also noted, resulting in the following subtypes: latero-lateral, antero-posterior, right-left coronary cusp fusion, right-non-coronary cusp fusion, and left-non-coronary cusp fusion [10].

More recently, Michelena et al. conducted an international consensus on the nosology of BAV, describing three main categories: complex valvulo-aortopathy, typical valvulo-aortopathy, and undiagnosed or uncomplicated form. Three principal phenotypes were also stated: fused BAV (right–left cusp fusion/RL, right-non cusp fusion/RN, left-non cusp fusion/LN, indeterminate cusp fusion), 2-sinus BAV (latero-lateral/LL, antero-posterior/AP), and partial-fusion BAV (fruste forms). In addition, aortic dilation was classified into root, ascending, and extended phenotypes [9].

### 3.2. Pathogenesis of Bicuspid Aortic Valve Disease-Associated Aortopathy in a Nutshell

Mechanistic processes underlying BAVD and associated disorders are complex and diverse, generating heterogeneous aortic phenotypes and variable clinical risk in these patients, with features distinct from those of TAV counterparts and with particularities of the pediatric population compared to adults.

A bicuspid aortic valve is macroscopically characterized by two asymmetrical functional leaflets, one of them having a midline raphe, or more rarely, two symmetrical cusps without a raphe, which predisposes to abnormal and turbulent flow patterns and higher tissue stresses [11]. The normal microscopic structure of the aortic valve consists of two central layers, the fibrosa (fibroblasts, collagen, and elastin fibers—with a role in strength and structural support), and the spongiosa (mesenchymal cells and proteoglycans—with a function in flexibility), and an endothelial surface [12]. Disorganized tissue architecture in the bicuspid aortic valve is linked to calcium deposition, leading to subsequent mineralization, fibrosis, and inflammation, which in turn determine aortic valve stenosis or regurgitation [11]. Aortic valve malformations trigger hemodynamic alterations predominantly in the ascending aorta, with secondary structural abnormalities of the aortic wall, thereby supporting the central role of hemodynamic factors in the etiopathogenesis of aortopathy associated with BAVD [13]. Nevertheless, structural abnormalities of the aortic wall commonly accompany BAV, even when the valve is hemodynamically normal, suggesting the possibility of premature dilatation of an already weakened aortic wall and highlighting the paramount influence of genetic predisposition in the development and progression of aortic dilation [11,13].

**Figure 2:**
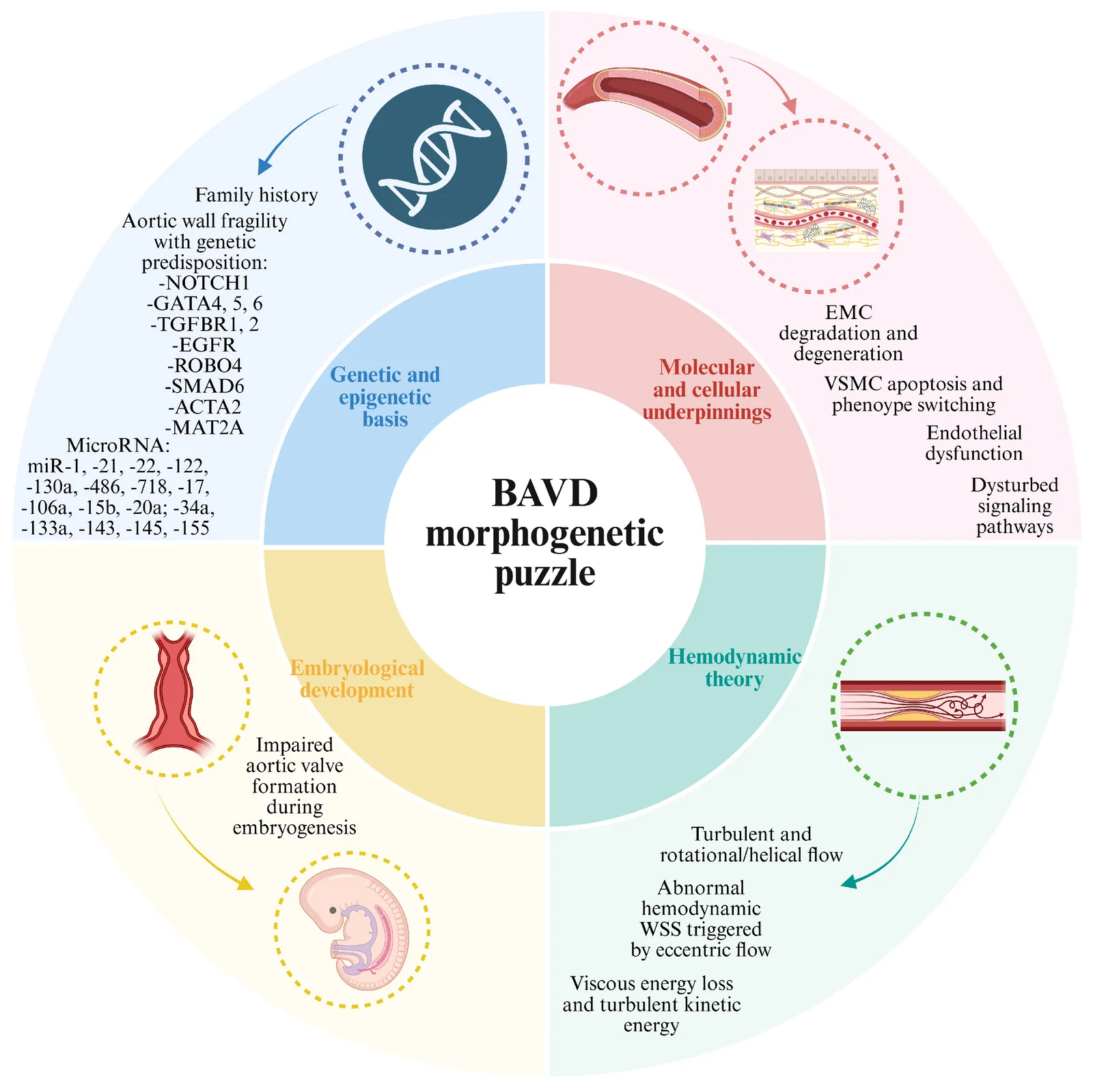
Bicuspid aortic valve disease morphogenetic puzzle. Created in BioRender. Man, O. (2026). https://BioRender.com/mjzlb6g (accessed on 29 August 2026). Abbreviations: BAVD, bicuspid aortic valve disease; EMC, extracellular matrix; VSMC, vascular smooth muscle cells; WSS, wall shear stress.

The interplay between genetic factors, molecular origins, and biomechanical and hemodynamic alterations has been extensively studied [12,14]. Taken together, these mechanistic insights form a complex puzzle in search of understanding the multiple possible origins of bicuspid aortic valve disease. We chose to classify and briefly describe these etiopathogenetic factors in the following sections, as illustrated in the image below: the embryological origin of the aortic valve and aorta; genetic and epigenetic hypotheses; cellular and molecular involvement; and hemodynamic theory (Figure 2).

Moreover, special attention must be given to the comparison between BAV and TAV aortopathy, as they differ not only in aortic valve structure but also in microscopic and macroscopic features of the aorta, derived biomechanical and hemodynamic behaviors, and clinical risk profiles [15].

Aortopathy associated with bicuspid aortic valve morphology arises from hemodynamic alterations characterized by asymmetrical, helical, and eccentric flow, with abnormal mechanical stresses in certain regions that affect the organization of the aortic wall and its biomechanical properties [16]. In some cases, the BAV-RL phenotype had higher frequencies of a dilated aortic arch and aortic stenosis than the BAV-AP phenotype [17]. Ascending aorta diameter is higher in BAV than in TAV patients, with mean values of 36.3 ± 6.3 mm vs. 31.4 ± 4.7 mm, according to adult echocardiographic reports [18]. Anatomy and geometry of the thoracic aorta are distinguished in relation to aortic valve morphology by some parameters, such as greater ascending aorta length and a lower angle between the aortic annulus plane and the plane passing through the top of the aortic commissures in BAV, according to the results reported by D’Ostrevy [19]. Regarding aortic regurgitation, a study by Yang et al. conducted on a large Asian cohort of adult patients found that a more severe aortic insufficiency was associated with a higher risk of aortic dissection (independently associated with aorta size ≥ 45 mm), and an earlier need for aortic surgery in TAV patients compared with BAV patients [20]. As for histological alterations, vascular development occurs in two phases—maturation starting from the prenatal period (intimal thickening) and degeneration, leading to weakening of the ascending aorta wall after birth in BAV, while three phases are described in TAV—maturation, stabilization, and degeneration, which may explain the elevated risk of bicuspid aortopathy [21]. Furthermore, several studies aiming to highlight distinct patterns between BAV and TAV aortopathy in adult subjects revealed an earlier age at onset of aortic dilation associated with BAV, thinner anterior and posterior aortic wall thickness in BAV samples, predicted by a dilatation > 51 mm and/or surface area/height ratio > 12, and slightly increased mechanical properties in BAV aorta influenced by a maximum aortic diameter > 52 mm and age > 66 years [22,23,24]. Biomechanical and elastic parameters were impaired in BAV as opposed to TAV aorta, with decreased aortic strain and distensibility and increased stiffness, according to the results found in a prospective study that included patients with ascending aorta dilatation with BAV (n = 33), or TAV with hypertension (n = 33), and 20 control subjects, with an overall mean age of 42.76 ± 10.4 years [25]. Interestingly, higher aortic dimensions and altered aortic elastic properties (strain, distensibility, stiffness) linked to elevated MMP-2 plasma levels were also observed in first-degree relatives of BAV patients, compared with TAV subjects, consistent with the genetic mechanism underlying BAV aortopathy [26]. Ten years after aortic valve or aortic surgery, valve morphology did not affect the rate of ascending aorta growth. Still, TAV phenotype was associated with a higher risk of adverse aortic events. In contrast, BAV was more frequently associated with a decreased risk of adverse aortic events after concomitant ascending aortic surgery, according to results from a single-center prospective observational cohort study of adult patients [27]. The American Association for Thoracic Surgery published consensus guidelines on bicuspid aortic valve-related aortopathy, covering epidemiological features, clinical presentation, diagnostic approaches, indications for familial screening, genetic testing, medical management, and surgical thresholds and follow-up [3].

The key aspects that differentiate BAV and TAV aortopathy, according to the aforementioned research in the field, are summarised in Table 1 below.

#### 3.2.1. Embryological Development of the Aortic Valve and Aorta

A defect in the early development of the aortic valve and ascending aorta wall might explain, to a certain point, the formation of a bicuspid aortic valve and congenital frailty of the aorta’s microscopic architecture, with an elevated risk of aortic dilation and acute aortic syndromes during lifetime [28]. After blastocyst formation, three germ layers of the embryo develop through epiblast migration and epithelial-to-mesenchymal transition: the ectoderm, mesoderm, and endoderm [29]. Epithelial-to-mesenchymal transition is mediated by transforming growth factor-β (TGF-β) signaling, Wnt (wingless-related MMTV integration sites), fibroblast growth factor (FGF), and the bone morphogenic pathway (BMP) [29]. The heart and vessels are the first organs formed during embryogenesis [28], starting from the differentiation of two mesodermal cell populations: the first heart field and the second heart field, into a myocardial scaffold that is the origin of the primitive heart tube [30]. Aortic valves, including the leaflets, develop from endocardial cushions in the atrioventricular canal and outflow tract, together with the involvement of two other distinct cardiac progenitor cells: the neural crest and the second heart field [28]. The aortic wall layers are formed from different cell types, as follows: neural crest cells for the tunica intima, second heart field cells for the tunica media containing vascular smooth muscle cells, and for the tunica adventitia [28]. A BAV phenotype may derive from defects during the following three valvulogenetic stages: endocardial cushion formation, outflow tract septation, or valve cushion excavation [30], caused by mutations in cardiac developmental genes, epigenetic alteration of such genes, dysfunctional variants in genes that may not be obviously linked to cardiac development, or even environmental factors [29]. However, BAV-associated genes may be precluded from discovery by various factors, such as the low frequency of individual variants, the high number of variants that cause BAV, variants with many structural types or functional roles, or mixed inheritance models of disease [29].

#### 3.2.2. Genetic and Epigenetic Basis

The genetic theory supporting BAV pathogenesis is documented partially by evidence of familial clustering and its presence in monozygotic twins and in one-third of newborns, with an autosomal dominant pattern of inheritance (47–89%) and male gender predominance, even if it is also characterized by incomplete penetrance, variable expressivity, and genetic heterogeneity [12,31]. The genetic hypothesis implicates genetic factors in intrinsic alterations of the aortic wall components, which create a favorable environment for aortic dilation [31].

Several gene mutations have been shown to be involved in different mechanistic steps driving the formation of bicuspid aortic valves and related conditions, as follows: (1) embryological formation of BAV (*GATA 4*, *5*, *6*, *NOTCH1*, *EGFR*, *TGFBR2*); (2) extracellular matrix remodeling (*BGN*, *COL1A1*, *2*, *COL3A1*, *EFEMP2*, *ELN*, *FBN1*); (3) vascular smooth cells apoptosis and switching phenotype (*ACTA2*, *MYH11*, *FOXE3*, *MAT2A*, *MYLK*, *PRKG1*); (4) TGF-β signaling pathways-related genes (*FBN1*, *NOTCH1*, *SK1*, *SLC2A10*, *SMAD2*, *3*, *4*, *TGFB2*, *3*, *TGFBR 1*, *2*) [31,32]. A study conducted by Luyckx identified seven novel loss-of-function and likely pathogenic variants of *SMAD6* from a total of 473 screened in BAV patients with thoracic aortic aneurysms [33]. Zheng investigated a potential promoter of aortic valve calcification in stenotic BAV samples, highlighting a SMAD-dependent signaling pathway partially responsible for low expression of the transcription factor SP2 in valvular interstitial cells and reduced expression of miR-195-5p [34]. Another study supported the contribution of *SMAD3* activation in BAV-associated ascending aorta dilatation, independent of TGF-β, pointing out its potential role as a therapeutic target [35]. Results reported by Musfee et al. identified 11 variants of *GATA4*, *SMAD6*, and *ROBO4* in 18% of BAV cases with early-onset complications, but they were not enriched in patients with thoracic aortic aneurysm or dissection [36].

While genetic mutations are static, epigenetic modifications are reversible and influenced by various environmental factors. They have gained growing recognition as contributors to thoracic aortic disease, with a potential utility in diagnosis and therapy [37]. MicroRNAs are small non-coding RNAs with a recognized role in various functional processes within the human organism, including the maintenance of ECM integrity and homeostasis by regulating gene expression post-transcriptionally through targeting messenger RNA (mRNA) transcripts for degradation or translational inhibition [37]. Regarding their role in BAV, Haunschild et al. showed that miR-29 is a crucial epigenetic regulator of ECM degeneration, being downregulated and associated with higher protein expression of MMP-2 in aortic tissue of BAV patients with aortic aneurysm [38]. In addition, specific molecules might enter the circulatory system, with involvement in various pathogenetic steps: malformation of the aortic valve (miR-1, miR-21, miR-122, miR-130a, miR-486), orchestration of aorta size (miR-718, miR-17, miR-106a, miR-15b, miR-20a), and vascular wall quality (miR-34a) [39]. In addition to miR dysregulation, *NOTCH1* hypermethylation has been implicated in the pathogenesis of BAV aneurysms, showcasing specific epigenetic fingerprints with the ability to distinguish BAV aortopathy from TAV-associated aneurysms [37].

A synthesis of the principal gene mutations and epigenetic factors that intervene in the etiopathogenesis of aortic dilatation in BAV patients is presented in Table 2.

#### 3.2.3. Molecular and Cellular Underpinnings

The aortic wall normally consists of three layers with distinct components and specific functions—tunica intima (endothelial cells arranged directly on the internal elastic lamina, in direct contact with the vascular lumen), tunica media (vascular smooth cells organized in concentric layers attached to the adjacent elastin–collagen extracellular matrix by fibrillin 1 microfibrils, which provide structural support, resistance, elastic function, and contractile properties in response to mechanical and chemical stimuli), and tunica adventitia (myofibroblasts producing collagen, with the capacity of dealing with stresses above physiological pressures) [8,15]. In contrast, histological analysis of the aortic wall of BAV patients revealed a disrupted morpho-architecture driven by different signalling pathways, with dysfunctional endothelial cells, cystic medial degeneration, and necrosis in the absence of inflammation, vascular smooth muscle cell immaturity, relaxation, apoptosis, detachment from the ECM, switching to a secretory phenotype and eventually death, deficiency in fibrillin 1, elastic fiber fragmentation, and, more recently, mucoid extracellular matrix accumulation [13,40]. The aortic media layer regulates tissue biology and biomechanics. Therefore, impaired structural integrity and dysregulation of this tunica, along with ECM abnormalities, are primarily responsible for the development and progression of bicuspid aortopathy, including ECM dysregulation by abnormal matrix metalloproteinase expression and activity, altered medial ECM architecture through elastin fiber degeneration, and tissue dysfunction through altered stiffness and biomechanics [41]. In a recent study, Bacour et al. [42] analyzed vascular wall tissue samples from premature infants to adulthood and described the histological lamellar architecture of the developing thoracic aorta. They also compared the aorta histology between BAV and TAV, based on age, demonstrating age- and morphology-related significant differences in the aortic wall organization, with the main findings being the higher number of elastic lamellae observed in TAV versus BAV samples, and the increasing number of lamellae from neonates to young children, followed by a gradual decrease from adolescents to older adults. Regarding blood flow patterns, laminar flow occurs through the normal TAV aorta, while helical blood flow in the BAV aorta results from a combination of ventricular twist and torsion during the systole, with eccentric outflow jet patterns disrupting laminar flow and flow impingement zones along the greater curvature of the ascending segment [43].

**Figure 3:**
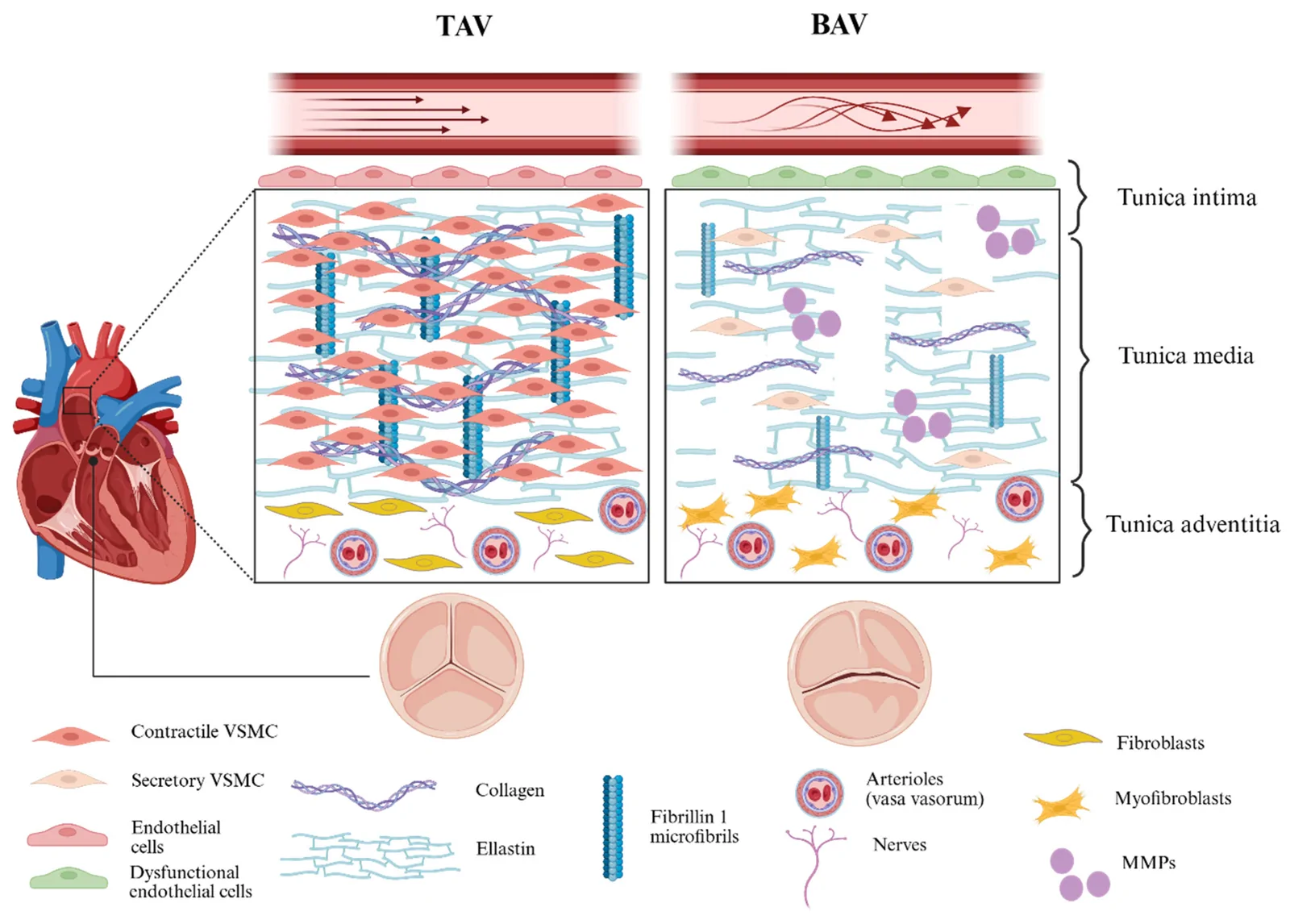
Microscopic architecture of the aortic wall and blood flow through the aorta—head-to-head illustrating a comparison between BAV and TAV aorta. Created in BioRender. Man, O. (2026) https://BioRender.com/84p7oi7 (accessed on 29 August 2026). Abbreviations: BAV, bicuspid aortic valve; TAV, tricuspid aortic valve; VSMC, vascular smooth muscle cells; MMPs, matrix-metalloproteinases.

The alterations in microscopic aortic wall architecture and flow patterns previously described in BAV are illustrated in the following figure by comparison with the TAV histological and hemodynamic profiles (Figure 3).

Understanding the molecular mechanisms underlying aneurysms associated with BAV is essential for early detection, identifying risk factors with prognostic significance for the development of complications, and optimizing therapeutic management. Thus, several studies have been conducted on this topic, with most of them focusing on proteolytic enzymes (matrix metalloproteinases—MMPs and their inhibitors—TIMPs), asymmetric dimethylarginine (ADMA), the soluble receptor for advanced glycation end-products (sRAGE), and transforming growth factor-β1 (TGF-β1), with a potential biomarker role in progression and involvement in the risk stratification of BAV-related aortopathy. Nevertheless, data in pediatric populations remain limited, and further longitudinal studies in larger cohorts are necessary to elucidate contradictory findings and establish their clinical applicability [5].

#### 3.2.4. Hemodynamic Theory

The hemodynamic hypothesis postulates that BAV-related aortopathy results from long-term valvular abnormality, with secondary blood flow disturbances and shear stress that alter intrinsic aortic wall structure, as evidenced by changes in the extracellular matrix and medial elastin fiber generation [31,44]. Many researchers have addressed this topic through experimental and imaging studies. A recent systematic review of the literature on this topic focused on 4D-magnetic resonance imaging and 3D-computational fluid dynamics for exploring blood flow patterns and relevant derived parameters through the aorta of BAV subjects, with three principal findings pointed out: helical and eccentric flow, correlation of flow displacement with valve morphology and aortopathy phenotype, and increased WSS along the greater curvature associated with aortic valve stenosis and peak systolic velocity in BAV-associated aortopathy compared to TAV-associated aorta [45]. Additionally, viscous energy loss and turbulent kinetic energy characterize the aorta related to BAV [32]. The modified hemodynamic features impact vascular biomechanical properties, as endothelial dysfunction triggered by WSS leads to production of nitric oxide and prostacyclin, as well as microRNAs inducing vasodilatation, promoting inflammation, and eventually driving arterial remodeling [32].

Notwithstanding, research is still needed to identify novel parameters for haemodynamic assessment in BAV aortopathy, especially in pediatric subjects and in conjunction with molecular studies, both of which are of utmost importance for understanding the etiopathogenesis and predicting future development and progression.

### 3.3. The Multifaceted Clinical Landscape of Pediatric Bicuspid Aortic Valve Syndrome

Without imaging evaluation of the aortic valve and aorta, most children may be completely asymptomatic, and the diagnosis is made after an echocardiographic assessment in the context of an incidental finding or family screening [7]. Even if it seems a silent disease, the underlying background of BAVD is not so benign, as symptoms that appear in youth or adulthood may reveal severe complications, even with fatal consequences [46]. A rigorous, scheduled program of continuous monitoring is therefore highly recommended for early detection of potential complications and associated disorders, and for optimal medical or interventional therapy [47]. First-degree relatives (FDR) screening of patients with BAV is also essential in current pediatric cardiology practice, given the genetic factors and the heritability demonstrated by Massardier et al. [48]. They prospectively included 213 consecutive index cases with BAV, with a median age of 11 years, and 713 patients identified as FDR, comprising parents, siblings, and offspring, with a mean of 3.3 ± 1.1 FDR per index case. They reported 6.6% of FDR having a BAV, 2.9% having ascending aorta dilatations based on Z-score, and 5.4% having aortic valve dysfunction [48].

The wide and heterogeneous spectrum of clinical presentations of BAVD in pediatric patients is partially based on the age at diagnosis and the predominant lesion (valvular or vascular). They can be classified into the following three main phenotypes, which are important for predicting future evolution and progression into adulthood: (1) asymptomatic normally functioning BAV phenotype; (2) aortic valve dysfunction phenotype, comprising congenital or acquired aortic valve stenosis and regurgitation; (3) primary aortopathy phenotype. Moreover, BAVD can be an isolated lesion or may be associated with other genetic disorders (Turner, Marfan, Williams, Loeys–Dietz, Ehlers–Danlos syndrome) or congenital heart diseases (coarctation of the aorta, supravalvular aortic stenosis, ventricular septal defect, Shone’s complex, interrupted aortic arch, hypoplastic left heart syndrome, tetralogy of Fallot, transposition of the great arteries) [8,46,47]. Asymptomatic patients who are evaluated for the first time by a pediatric cardiologist may present with a systolic ejection click and sometimes a soft systolic ejection murmur [7,47].

As for age differences in clinical presentations of BAVD, severe aortic stenosis presents as cardiogenic shock in neonates or as progressive congestive heart failure in infants, while symptoms at older ages are absent or mild (fatigue, dyspnea, syncope, chest pain, angina). Aortic regurgitation is rare in infants, whereas severe forms in older children and adolescents may present with fatigue, dyspnea, or limited exercise capacity. Aortic dissection is also very rare across all age groups and is associated with other genetic or connective tissue disorders. Clinical symptoms include acute onset and sharp, tearing chest pain radiating to the back, neck, or abdomen [7].

Regarding gender differences among BAV patients, men are known to be mainly affected, but they are also more predisposed to develop moderate/severe aortic regurgitation earlier and to present with a larger aortic diameter at the sinus of Valsalva level, while women commonly experience aortic stenosis [49,50].

Baseline aortic dilatation ≥40 mm, the severity of aortic regurgitation or stenosis, and age are predictors of cardiac events in BAVD. In a cohort of 581 consecutive cases with a median age of 29 years, 27% of patients experienced an adverse event within 5 years [51]. Furthermore, ascending aorta dilation and aortic stenosis are more commonly linked to the RN-BAV phenotype, while dilation of the aortic root at the sinuses of Valsalva is more often observed in the RL-BAV phenotype among young patients [52]. The aortic dilatation phenotype may influence the risk of developing aortic dissection, as documented in a large retrospective cohort study recently published. The study showed that adult BAV patients with aortic root dilatation exhibited faster aortic growth of the aortic root (0.86 ± 1.82 vs. 0.77 ± 1.90 mm/year) and the ascending aorta (0.93 ± 2.12 vs. 0.79 ± 2.04 mm/year, *p* < 0.001), with over a two-fold increased risk of aortic dissection (AUC = 0.752) compared to patients without aortic root dilatation [53]. Factors predicting aortic dilation therefore play a crucial role in the management of patients with BAV. Data provided from the analysis of a cohort comprising 279 BAV patients aged between 17 and 30 years, with moderate to early onset severe valvular or aortic disease revealed that almost 30% presented with associated congenital heart malformations, with higher prevalence of aortic regurgitation and RN phenotype, 50% required surgical intervention in childhood and 25% in young adulthood, aortic valve replacement or repair was indicated in 44% of cases, reinterventions occurred in 22% of cases mainly because of aortic aneurysm, underlying the need for prompt recognition and strictly scheduled monitoring, to ensure optimal therapy before severe complications develop [54].

An interesting finding has been reported by Nadorlik et al. [55], who showed that children with familial BAV have a propensity to develop BAV and abnormal aortic growth, with a higher risk of ascending aortic dilatation in the RL-BAV phenotype, supporting the need for familial screening and potential initiation of medical treatment to prevent aortopathy in childhood.

In the pediatric population with isolated BAVD, the incidence of primary cardiac events defined as surgery on the aortic valve or ascending aorta, percutaneous valvuloplasty, aortic dissection, infective endocarditis, or cardiac-related death is relatively low (0.004 per patient-year), approximately 3-fold lower than in young adults. Events are mainly aortic stenosis with an indication for balloon dilatation, and aortic valvular dysfunction progresses relatively slowly. Mild ascending aorta enlargement commonly associated with a higher grade of aortic regurgitation is frequent, but has a relatively benign course (Z-score increases by 0.1/year) and carries no significant risk of aortic dissection [56,57,58]. Even a functionally normal BAV (peak gradient ≤ 16 mmHg) is complicated by progressive aortic dilatation beginning in early childhood, with aortic diameter increased by approximately 1 SD every 5 years and the most accelerated growth rate of the ascending aorta dimensions compared to TAV patients (2.3 ± 0.6 vs. 1.8 ± 0.5 cm, *p* < 0.0001), predicted by the RN phenotype and the initial aortic valve gradient (>15 mmHg) [59,60], suggesting the importance of serial echocardiographic evaluations. More recent studies have confirmed an overall slow and clinically non-significant rate of proximal aorta dilatation in children with a normally functioning BAV, with an increase in ascending aorta Z-score of 0.06–0.09 units per year between ages 5 and 15 years and a higher risk when associated with aortic valve stenosis or insufficiency [61,62]. In addition, mixed aortic valve lesions, higher aortic distensibility, lower aortic stiffness, and RN cusp fusion have potential utility to predict aortic dilatation in BAV children and therefore should be closely monitored during long-term follow-up, even if significant ascending aorta dilatation may occur in the absence of aortic valve stenosis or insufficiency [63,64,65]. However, based on the previous studies mentioned in this section, there are still controversies regarding the indications of early medical treatment or elective surgery in children with BAV and significant aortic valve disease and their potential to prevent associated aortopathy progression.

### 3.4. Biomarkers of Pediatric Bicuspid Aortic Valve Disease-Associated Aortopathy—Addressing Small Pieces of a Large Puzzle

Extensive research has explored the potential roles of various blood biomarkers in BAV-aortopathy, primarily because these biomarkers are easily accessible and cost-effective. This makes them suitable for diagnostic use and potentially valuable for prognostic staging and prediction models, especially compared with imaging-based size measurements. Notably, interest has increased in certain biomarkers, including protein-based markers (such as matrix metalloproteinases, tissue inhibitors of metalloproteinases, transforming growth factor-β, alpha-1 antitrypsin, and advanced glycation end products) and genetic or epigenetic markers (including non-coding RNAs such as microRNAs). However, few studies have included pediatric patients, keeping this an ongoing area of interest and concern, and a promising avenue for future research [66,67,68].

Pediatric BAV aortopathy biomarker research faces limitations due to scarce pediatric-specific data, small single-center samples, and absence of longitudinal validation, preventing any circulating biomarker from being ready for clinical risk assessment in children [5]. Most candidate biomarkers, such as MMPs, TIMPs, TGF-β, and microRNAs, have been studied primarily in adult populations, with only a few pediatric studies available, mostly exploratory [5].

The subsequent sections will provide a brief overview of circulating biomarkers, focusing on the biological mechanisms underlying bicuspid aortopathy.

(a) Extracellular matrix remodeling

The imbalanced secretion of MMPs and their inhibitors, TIMPs, in response to turbulent flow and shear stress is recognized as a significant contributor to ECM remodeling, driven by the loss of elastic fibers and collagen degradation, which appear to be hallmarks of progressive aortic enlargement [69]. Tissue and circulating levels of MMP-2 and MMP-9 are the best-examined and have been reported to be higher in patients with BAV than in patients with TAV [15]. Significant linear correlations between specific microRNAs (miR-133a, miR-143a) and MMP-2, TIMP-1, TIMP-2 protein expression at the level of the greater curvature aortic tissue were found in patients with BAVD and aortopathy, suggesting that the impact of microRNA regulation on MMPs/TIMPs might be involved in the development of ascending aorta remodeling [70]. Different expression of microRNAs and MMP/TIMP between the greater and lesser curvatures of the ascending aorta in BAV patients appears to be influenced by rheological factors such as WSS, supporting the interconnection of molecular and hemodynamic mechanistic pathways in the formation of aortopathy [70]. Moreover, even though plasma levels of MMP-1, MMP-8, MMP-9, MMP-10, and TIMP-1 were higher in isolated severe BAV stenosis as opposed to healthy patients, only elevated MMP-9 expression distinguished between patients with and without aortic dilatation, whereas MMP-2 levels were higher in aortic dilatation BAV patients, independently of the aortic stenosis status, as stated by Wang et al. [71]. Interesting results were reported by Schmitt [72], who discovered significantly higher tissular levels of Pro-MMP-2, total MMP-2, and TIMP-2 in the posterior part of ascending aorta aneurysms associated with BAV compared to the anterior part, which might be partly explained by differences in hemodynamic features between the two distinct regions of the aorta. Similar findings were documented by analyzing tissue or circulatory levels of MMPs/TIMPs, showing higher expression of MMP-2 [73,74,75], MMP-9 [76,77] in dilated aortas associated with BAV than in those with TAV, also depending on aortic dimensions, elastic and biomechanical characteristics, and aortic valve morphology in some cases.

In contrast to previous studies on adult populations, pediatric research is still scarce. Recent studies conducted by Fagarasan in 2024 [78] and Senturk in 2026 [79] draw our attention to circulating biomarkers and aortic biomechanics in pediatric BAV cohorts. In a separate observational cross-sectional study of 73 children with BAV, Făgărășan et al. found that among MMP-1, MMP-2, MMP-9, and TIMP-1, only TIMP-1 was significantly higher in BAV controls without dilatation and was inversely correlated with aortic annulus and ascending aorta Z-scores. MMP-1, MMP-2, and MMP-9 did not differ between dilated and non-dilated groups [78]. In a prospective case–control study of 40 children with BAV versus 40 healthy controls, serum levels of MMP-2, MMP-9, and the MMP-2/TIMP-1 ratio were significantly elevated and associated with impaired aortic strain and distensibility. However, biomarker associations were less consistent in analyses restricted to the BAV group [79].

Taken together, these results shed light on the altered architecture of the aortic wall in BAV subjects exposed to specific flow and shear stress conditions, resulting in impaired MMP and TIMP secretion. These changes may serve as promising biomarkers for the progression of aortic dilation in BAV aortopathy. However, discrepancies across studies indicate that data on pediatric MMPs and TIMPs remain inconsistent, and long-term research from early childhood is necessary to determine their predictive value.

(b) Endothelial dysfunction and signaling

Beyond MMPs and TIMPs, several novel biomarker classes show early potential. The TGF-β superfamily plays a crucial role in aortic vascular remodeling and endothelial dysfunction [16]. A significantly thinner intimal layer, defective TGF-β signaling in the tunica intima, immature VSMCs, loss of TGF-β expression in the tunica media, and differences in vasa vasorum in the tunica adventitia characterize the histology of the aorta associated with BAV [80]. Lower levels and muted activation of TGF-β1, with latent TGF-β binding protein 3, were reported by some authors in adult patients with BAV compared with TAV and in monogenic forms of thoracic aortic aneurysms (Marfan and Loeys-Dietz syndromes) but were not associated with aortic dilatation [81,82]. In contrast, higher TGF-β2 expression was observed in both BAV- and TAV-associated aortas and was positively correlated with aortic diameter in the BAV convexity [83]. The serum TGF-β1/soluble endoglin ratio uniquely increased in BAV patients (not tricuspid valve patients), reflecting gene expression changes observed in the aortic tissue (increased MMP-2 and TGF-β1 expression and decreased MMP-14 and superoxide dismutase 3), and correlated with future aortic growth rate, representing a possible early prognostic marker, though validation in pediatric-specific cohorts is lacking [84].

TGF-β1 levels did not differ between pediatric BAV and TAV patients, but showed modest correlations with aortic biomechanics within the BAV cohort, according to Senturk et al. [79]. Furthermore, distinguishing BAV children with and without aortic dilatation might be guided by plasma miR-130a expression, which is decreased in patients with aortic dilatation, and by targeting genes involved in the TGF-β signaling pathway, which is implicated in endothelial dysfunction [85].

MicroRNA profiling has identified promising biomarker candidates for BAV-associated aortopathy, but the evidence base is limited by small cohort sizes, inconsistent validation, and unresolved questions regarding tissue specificity [66,86]. Circulating microRNAs, particularly miRNA-17, and desmosine, when combined with imaging evaluation, show promise in adult BAV populations, given their stability in both tissues and blood, but single-miR prediction models have not achieved sufficient accuracy for BAV-associated aortic aneurysms. Moreover, they have not been studied in children [39,66]. Dysregulation of miR-424-3p and miR-3688-3p in both aortic tissue and blood was documented in patients with BAV-related aneurysms, thereby impairing SMAD7 expression in the TGF-β signaling pathway [86]. Together with miR-34a, miR-125a modulates MMP-2 by suppressing its expression in aortic wall tissues exposed to high wall shear stress and contributing to ECM remodeling, according to the results reported by Lu et al. [87]. Moreover, five microRNAs (miR-128-3p, miR-210-3p, miR-150-5p, miR-199-5p, miR-21-5p) have different expression across dilated ascending aorta circumference (anterior/right wall segments), concomitantly with decreased elastic fibers and elevated WSS approached by 4D-CMR scans [88]. Recently, the expression patterns of seven molecules of microRNA (miR-17, miR18a, miR-19a, miR-20a, miR-21, miR-106a, miR-145) were quantified from serum of BAV patients, resulting in significantly different profiles in comparison with TAV, and a negative correlation with maximal aortic diameter, suggesting potential utility for future risk stratification, although independent validation in large longitudinal cohorts is required [89].

Nonetheless, research on miRNA in bicuspid aortopathy remains preliminary and lacks essential validation, arising mainly from methodological and biological gaps and limited translational readiness. Major limitations include small sample sizes and single-center cohorts, which limit statistical power [16,88]; poor NGS-qPCR concordance [86]; no aortopathy-specific molecules [70,89]; unproven blood-tissue correlation [70]; and lack of functional validation [86]. In particular, the absence of an aortopathy-specific miRNA remains a fundamental barrier. Circulating miRNAs indicate various systemic conditions—such as malignancy, inflammation, and cardiomyopathy—making their specificity for aortic wall remodeling uncertain [67,70]. It has not been definitively shown whether circulating miRNAs linked to progressive aortopathy originate from aortic tissue rather than other systemic sources, due to factors such as heterogeneity in BAV phenotypes, confounding comorbidities that affect miRNA levels, and ongoing debates about the direction of dysregulation among multiple miRNAs [16,88,89]. Multiple studies explicitly frame their findings as preliminary and hypothesis-generating rather than clinically actionable, given that miRNA derangements have limited practical impact and require larger, well-defined BAV cohorts for validation [66,86,89]. Whether circulating miRNAs are directly involved in aortic wall pathogenesis or are a consequence of increased aortic shear stress from anomalous BAV flow remains unknown [85]. This causal ambiguity—combined with the lack of multicenter replication, standardized miRNA panels, and mechanistic functional studies—firmly places miRNA biomarker development for bicuspid aortopathy in the discovery phase.

(c) Systemic inflammation

Systemic inflammatory indices, including the monocyte-to-lymphocyte ratio (MLR), systemic inflammatory response index (SIRI), and pan-immune-inflammation value (PIV), might classify BAV children with and without ascending aortic dilatation, suggesting potential involvement of inflammation in the pathogenesis of aortopathy [90]. Similarly, the MLR independently predicted Stanford type A dissection (HR 8.87) in a large BAV cohort, though this study was not pediatric-specific [53].

Despite the growing literature in the field, several limitations currently preclude clinical validation and applicability of circulating biomarkers in pediatric bicuspid aortopathy and should be acknowledged. Firstly, most biomarker research focuses on adults, and pediatric-specific studies are rare and often yield contradictory results, with MMP studies showing inconsistent findings across cohorts. Also, age-dependent reference ranges have not yet been established, further limiting pediatric applications, as physiological levels in children differ from those in adults [5,78]. Moreover, the z-score used to define aortic dilatation in children may overstate the incidence of dilatation, especially in adolescents, potentially misclassifying biomarker associations [78]. The most striking gap is the near-complete absence of multicenter prospective pediatric biomarker studies. No candidate circulating biomarker has been serially measured or followed up long-term in children to demonstrate that early elevation predicts future aortic events, including aneurysm formation or dissection [66]. Most studies were conducted on small, single-center samples: the largest pediatric circulating biomarker study enrolled 73 children at one center, whereas the only integrated imaging-biomarker pediatric study included 40 cases [78,79]. Although genetic and epigenetic markers, such as rare variants and methylation signatures, are emerging, they are still investigational. Combining functional imaging with circulating biomarkers through integrated approaches could improve early detection beyond traditional diameter-based monitoring [79].

In summary, research on biomarkers for pediatric BAV aortopathy remains in an early exploratory stage, constrained by limited data and small study sizes. Circulating MMPs, TIMPs, and molecules of the TGF-β pathway are the most promising candidates but have yet to demonstrate proven clinical utility [78,79]. Most biomarkers studied in pediatric populations are unconfirmed, and no single marker is currently approved for risk assessment [5,66]. The field needs longitudinal studies with repeated biomarker sampling in pediatric cohorts to determine causal links between circulating markers and meaningful aortic outcomes [66]. Although these circulating biomarkers provide valuable insights into the biological mechanisms underlying pediatric BAV-related aortopathy, they remain experimental and are not yet part of routine clinical practice. At present, they do not replace standard monitoring of aortic size and valve function, and biomarker assessment remains a research tool rather than a clinical standard.

### 3.5. Multimodal Imaging Evaluation of Pediatric Bicuspid Aortic Valve Disease and Associated Conditions—Focusing on the Aorta

The study by Grattan et al. in the multicenter MIBAVA project explored the relationships among valve phenotype, valvular dysfunction, and ascending aortic dilatation in children with BAV. Of 2122 pediatric BAV patients, half had ascending aortic dilatation. Factors like right-noncoronary cusp fusion, more severe aortic stenosis and regurgitation, along with older age, were independently linked to this dilatation. Interestingly, 37% of patients without any aortic stenosis or regurgitation also exhibited dilatation. These results suggest that valve-related hemodynamic factors play a role, but aortic dilatation can occur even without clear valvular issues, supporting a multifactorial model of pediatric BAV aortopathy [64].

The evolving landscape of multimodal noninvasive imaging techniques offers valuable tools and novel parameters for a thorough evaluation of BAV-related aortopathy, with diagnostic and prognostic impact. Current guidelines for the management of aortopathies in pediatric and adult patients recommend an initial imaging evaluation of the aortic valve and thoracic aorta using transthoracic echocardiography, in combination with computed tomography or magnetic resonance imaging in selected cases, especially when ultrasound does not allow optimal visualization and quantification of key parameters for assessing aortopathy or after aortic surgery [3,6,91].

#### 3.5.1. Transthoracic Echocardiography

Transthoracic echocardiography (TTE) is the first-line imaging tool used to diagnose BAV, classify its morphology and monitor related aortopathy in children, achieving a sensitivity of 92% and a specificity of 96% [2,57,92].

Population screening at birth shows BAV occurs in about 0.8% of cases [93,94]. Family screening echocardiography of first-degree relatives yields a threefold higher prevalence of BAV and/or aortopathy compared with controls, supporting echocardiographic screening of relatives [93]. Additionally, focused imaging protocols may facilitate screening for BAV in specific populations. However, a thorough echocardiographic assessment remains essential when a detailed evaluation of valve morphology and function, associated congenital anomalies, or aortic measurements is needed [95].

Aortopathy, defined as an aortic diameter z-score of ≥2 or ≥3 depending on the study, is common in neonates and young children with BAV. Its prevalence in pediatric groups ranges from 33% to 50% [64,94,96]. Standard pediatric BAV echocardiography requires assessment of the entire thoracic aorta at four levels: the annulus, sinus of Valsalva, sinotubular junction, and proximal ascending aorta [55,57]. Aortic diameters are indexed to body surface area using pediatric nomograms and expressed as z-scores. Measurements are taken using the leading-edge-to-leading-edge method in end-diastole, with the annulus measured from inner-edge to inner-edge in systole [57,78]. Pediatric cardiologists use different Z-score thresholds for mild (mean 2.13), moderate (3.59), and severe (5.11) aortic root dilation, with the Boston and Pediatric Heart Network (PHN) systems being the most commonly used [97]. Traditional Z-scores identified ascending aorta dilation in 31–41% of isolated BAV cases, compared to only 15–16% with the Q-score, indicating potential overestimation by standard nomograms [57]. The PHN nomogram reports higher ascending aorta Z-scores than the Halifax nomogram (median 1.52 vs. 1.41), with strong correlation (ρ = 0.979), but differing results at extreme Z-scores [98]. In adolescents (BSA ≥ 1.5 m^2^), BSA-indexed thresholds classify fewer patients as dilated than Z-score nomograms; for aortic root, the classification is not affected by the reference used [98,99]. This variability leads to differing interpretations and management strategies, such as restrictions on competitive sports, even when standardized cutoffs are applied [97]. No cases of aortic dissection or preventive surgery have been reported in pediatric BAV cohorts with longitudinal Z-score monitoring [58,62,64]. In children with BAV, aortic diameter Z-scores serve as a body-size-adjusted metric to monitor aortopathy. However, the selected nomogram significantly impacts prevalence estimates—especially in adolescents nearing adult body size. In this group, traditional Z-scores may overstate dilation, whereas BSA-indexed thresholds provide a more cautious alternative.

TTE also enables the quantification of biomechanical and elastic aortic parameters (strain, distensibility, and stiffness indices) [100,101,102,103]. Children with BAV demonstrate impaired aortic elastic properties—including lower aortic strain, reduced distensibility, and higher stiffness index—before overt aortic dilatation or myocardial dysfunction [79].

Moreover, blood flow visualization and quantification reveal hemodynamic disturbances in the proximal aorta and left ventricle, even in the absence of significant aorta enlargement, which suggests a potential to predict future aortic dilatation and left ventricular diastolic dysfunction, but future longitudinal studies on larger cohorts are required for clinical validation [104]. Beyond diameter-based assessment, echocardiographic pulse wave velocity (PWV) measurement reveals increased aortic stiffness in children with BAV even when conventional distensibility parameters are normal [64]. Aortic arch PWV is the only vascular function parameter independently associated with aortic dilatation in multivariate analysis [105]. Conventional diameter-based elasticity parameters may appear normal in children with normal-functioning BAV, while echocardiographic PWV measurements detect significantly increased aortic stiffness [106].

A summary of the main studies assessing hemodynamic alterations through echocardiography in pediatric BAV-aortopathy is presented in Table 3 [104,106].

Valve morphology is classified from the parasternal short-axis view, using the Sievers or Michelena system to identify the number of raphes and cusp orientation [57,94,107]. Right-left cusp fusion is the most common morphology (65–75%), associated with coarctation and root dilation [4,57,94]. Right-noncoronary fusion is linked to valve dysfunction (stenosis and/or regurgitation) [108]. Aortic coarctation must be excluded via suprasternal and abdominal aortic views in every BAV evaluation [108]. The severity of aortic valve stenosis is primarily assessed using Doppler-derived transvalvular peak velocity and pressure gradients [109]. Evaluation of aortic valve regurgitation requires an integrated multiparametric approach that incorporates CD, vena contracta, flow reversal, and ventricular remodeling [109]. In children with BAVD, recent echocardiographic findings demonstrate a higher prevalence of aortic valve stenosis or insufficiency and a higher aortic sinus Z score in RN than in RL phenotypes [110], greater stiffness and lower distensibility in the ascending and abdominal aortas and carotid arteries, and higher carotid intima–media thickness values in BAV than in TAV patients [111].

Left ventricular structure and diastolic and systolic myocardial function are characterized using two-dimensional (2D), color Doppler (CD), blood-pool and tissue pulse-wave (PW) Doppler, and conventional continuous-wave (CW) Doppler, with M-mode measurements from the parasternal long-axis view, normalized for age, body mass index and body surface area [112]. Tissue Doppler enables assessment of myocardial function by measuring longitudinal peak mitral annulus velocities during systole (S′), early (E′), and late (A′), which are measured throughout the cardiac cycle at the mitral lateral annulus and at the basal septal wall in apical 4-chamber views [109]. Left ventricular end-diastolic diameter, left ventricular end-systolic diameter, left ventricular posterior wall thickness, and left atrial diameter are measured, and ejection fraction and fractional shortening are further calculated from M-mode echocardiographic tracings [113].

Novel echocardiographic techniques enable assessment of left ventricular myocardial deformation via speckle-tracking analysis, revealing a decline in global longitudinal strain (GLS) that can indicate clinical heart failure even before ejection fraction decreases [107].

Therefore, TTE is the technique of choice for the initial diagnosis of BAV and assessment of valvular function [2,92,108]. It remains accurate and reliable for evaluating BAV morphology and aortopathy in children [96]. However, it may underestimate the severity of aortic dilatation by 5–7% compared with CMR, with larger systematic differences in BAV due to root asymmetry [96]. CMR detects severe aortic dilatation in 54% of patients versus 38% by echocardiography, and BAV morphotype classification differs significantly between the two modalities [96]. Identification of a raphe can be particularly challenging by echocardiography, potentially leading to morphotype misclassification [96].

**Table 3:** Summary of main findings from studies analyzing blood flow disturbances in pediatric BAV-associated aortopathy.

**Ref.**	**Author**	**Year**	**Aim of the Study**	**Imaging Tool**	**Main Results**	**Limitations**
[104]	Henry	2023	- To assess qualitatively complex flow in the aortic root and left ventricle in children with BAV compared to TAV - To quantify differences in flow dynamics	Blood speckle tracking 2D-ultrasound	- 38 participants: 14 BAV, 24 TAV - Aortic root analysis: - BAV: eccentric and less organised flow vs. TAV: more laminar and unidirectional flow - BAV: peak VO and KE are more variable and eccentric in location vs. TAV: high VO in the sinuses and a central stream of high KE - BAV: increased peak systolic rate of EL and VC vs. TAV (15.9 ± 7.5 mW/m vs. 9.1 ± 8.6 mW/m and 0.53 ± 0.19 vs. 0.24 ± 0.20) - Left ventricle analysis: - BAV: increased peak diastolic KE (0.18 ± 0.08 J/m vs. 0.08 ± 0.05 J/m), rate of EL (28.7 ± 15.11 mW/m vs. 6.8 ± 4.7 mW/m), VO (39.8 ± 11.6 Hz vs. 22.7 ± 7.8 Hz), and VC (0.70 ± 0.12 vs. 0.57 ± 0.18) vs. TAV	- 2D nature of flow analysis - relatively small number of patients - limited conventional echocardiographic data in the control group - technical limitations with a focus on flow dynamics in children <10 years of age and in the aortic root
[106]	Ertaș	2026	- To measure aortic local PWV without requiring any specialized software or equipment - To assess the reliability of this measurement method	Transthoracic echocardiography	- 50 BAV children, 50 healthy children - higher peak aortic velocity in BAV - higher aorta diameters in BAV - significantly higher PWV in BAV - positive correlation between PWV and aortic root diameters, peak aortic velocity, vena contracta in aortic regurgitation, and time to reach the descending aorta	- single-center study - small number of patients - intermittent measurement of the distance between the ascending and descending aortas in PWV quantification - measurement errors - PWV is affected by blood viscosity
[114]	Meierhofer	2013	- To compare flow patterns and WSS in the ascending aorta of BAV vs. TAV patients	4D-flow CMR	- 18 BAV patients, 18 controls - BAV: 85% grade 2 or 3 blood flow patterns (helical) - TAV: 94% grade 0 or 1 blood-flow patterns - BAV: axial, circumferential, and magnitudinal WSS is significantly abnormal in the mid-ascending aorta at the level of the main pulmonary artery, compared with TAV - BAV: decreased axial WSS and increased circumferential and magnitudinal WSS compared with TAV - BAV: in the distal ascending aorta at the level just before the branching of the brachiocephalic trunk, only circumferential strain was significantly increased compared with TAV	- histological alterations in the aortic wall not examined
[115]	Allen	2015	- To describe the influence of BAV on thoracic aorta hemodynamic parameters in a group of pediatric and young adult patients	4D-flow MRI	- 30 BAV patients, 17 with aortic root dilatation and 14 with ascending aorta dilatation - positive correlation between mean and maximum systolic WSS and peak systolic velocity in the ascending aorta (r = 0.84, *p* < 0.001; r = 0.94, *p* < 0.001) - the cohort average echo-measured peak velocity was significantly higher than 4D flow-measured peak velocity (2.1 ± 0.98 m/s vs. 1.27 ± 0.49 m/s, *p* < 0.001). - the indexed aortic root diameter growth rate: −0.9 ± 2.5 mm/m^2^/year - the absolute aortic root diameter growth rate: 1.1 ± 1.4 mm/year - the indexed ascending aorta diameter growth rate: −1.2 ± 1.73 mm/m^2^/year - the absolute aortic root diameter growth rate: 1.1 ± 1.2 mm/year - no significant correlations between aortic growth rates and systolic WSS	- potential selection bias (BAV population already sent for MRI) - velocity data at the wall and calculated WSS may be more impacted by partial volume effects than a similar study in adults with larger anatomy. - the use of time-averaged segmentations to study WSS - the regional variation in WSS within the ascending aorta was not explored, which may have been impacted by aortic diameter or valve morphology. - lack of normal controls - lack of gold standard measure of WSS
[116]	Rose	2019	- To investigate whether hemodynamic parameters change over time in children and young adults with BAV	4D-flow MRI	- 19 BAV patients - similar aortic root and ascending aortic Z-scores at baseline and follow-up (aortic root: 3.25 ± 1.81 vs. 3.45 ± 1.91, *p* = 0.44, mean annual change = 0.2 ± 0.6 per year; ascending aorta: 3.12 ± 2.62 vs. 3.59 ± 2.76, *p* = 0.08, mean annual change = 0.6 ± 0.6 per year) - no significant changes in peak systolic velocity in any aortic regions for the full cohort or for any of the subgroups. - no significant regional changes in 3-D WSS for the full cohort	- short-term follow-up - retrospective study - relatively small cohort
[117]	Stefek	2019	- To provide a comprehensive evaluation of global LV function, myocardial strain and aortic flow hemodynamics in children with BAV compared to TAV controls - To quantify the impact of valvular stenosis and regurgitation on these parameters	CMR	- 58 BAV children, 25 TAV controls - no significant difference in ejection fraction between the study groups - similar peak velocity and WSS between the BAV subgroup without valve disease and controls - lower GLS in post-surgical patients than in controls	- small sample size - relative lack of young children
[118]	Stefek	2020	- To quantify aortic root asymmetry, or eccentricity, in adult and pediatric BAV patients and TAV controls - To observe the - relationship between sinus eccentricity and presence of valvulopathy or aortopathy	CMR	- 149 adult BAV patients, 51 pediatric BAV patients - BAV: 65% of patients with RL fusion demonstrated qualitatively dominant N sinus and 81% of patients with RN valve fusion demonstrated qualitatively dominant L sinus - TAV: 20% of TAV controls had a qualitatively dominant sinus, with variable position of the largest sinus - similar pattern of sinus eccentricity between children and adults - aortic stenosis: 18 RL adults (15%), 5 RN adults (18%), 8 TAV adults (20%), 11 RL children (27%) - aortic regurgitation: 52 RL adults (43%), 11 RN adults (39%), 15 TAV adults (37%), 12 RL children (29%), 3 RN children (30%)	- lack of young children - differences in adult and pediatric imaging protocols
[96]	Krasic	2024	- To compare the TTE and CMR findings in patients with BAV - To define valve dysfunction and aortopathy risk factors	TTE vs. CMR	- 50 BAV pediatric patients - the BAV morphotypes were evaluated significantly differently by TTE and CMR - BAV insufficiency was more frequently observed by TTE than CMR - BAV stenosis was found in 44% of patients by TTE and 58% by CMR - aortopathy frequency rate: 76% by TTE, and 78% by CMR	- retrospective study design - small sample size
[119]	Fujiwara	2024	- To investigate if abnormal directionality of blood flow obtained from 4D flow CMR is associated with aortic dilation in pediatric patients with BAV	4D-flow CMR	- 53 BAV pediatric patients (18 with associated coarctation of the aorta) - significantly higher mean velocity at distal ascending aorta in patients with CoA-BAV - significantly larger normalized helicity density at the mid and distal ascending aorta in CoA-BAV group - severely abnormal direction more often seen in the BAV group for both velocity and WSS at the mid ascending aorta - moderately abnormal direction of velocity more commonly seen in the BAV group at the distal ascending aorta - significant differences in the severely abnormal direction of velocity at the distal ascending aorta	- retrospective study design - limited number of controls

Abbreviations: Ref., reference; VO, vorticity; EL, energy loss; KE, kinetic energy; VC, vector complexity; PWV, pulse wave velocity; 4D-flow CMR, four-dimensional cardiovascular magnetic resonance imaging; 4D-flow MRI, four-dimensional flow magnetic resonance imaging; WSS, wall shear stress; RN, right–noncoronary cusp fusion; RL, right–left cusp fusion; TTE, transthoracic echocardiography; CoA-BAV, coarctation of the aorta associated with bicuspid aortic valve.

#### 3.5.2. Computed Tomography

Compared to cardiac ultrasound, cardiac computed tomography (CT) is especially advantageous because it has a high diagnostic performance in BAV and associated disorders, playing an important role in the diagnosis of concomitant disease, as it provides an accurate anatomical description of cardiovascular structures and allows calculation of various parameters, as follows: aortic valve orifice area, calcium score, etc [120]. Computed tomography angiography (CTA) and derived 3D programs offer novel insights with promising applications in clinical practice for anatomic reconstruction, complementing computational flow analysis used to evaluate hemodynamic alterations associated with bicuspid aortopathy [121].

#### 3.5.3. Cardiac Magnetic Resonance Imaging

Transthoracic echocardiography continues to be the primary method for assessing pediatric BAV aortopathy and for serially monitoring aortic dimensions in children [92,96,122]. However, additional cardiac magnetic resonance imaging (MRI, CMR) provides superior accuracy and reproducibility for the aortic root and ascending aorta, especially when asymmetry is present, and may identify severe aortic dilatation more frequently (54% vs. 38%) [92,96,122]. This indicates that using multiple imaging modalities could be beneficial in specific cases. Incorporating multiparametric CMR evaluations—considering aortic size, stiffness, and 4D-flow hemodynamics—may improve risk stratification for cardiac remodeling and dysfunction in pediatric BAV patients [117].

As for the assessment of left ventricular systolic function through GLS analysis, which is a sensitive marker of myocardial deformation, it is commonly performed by using speckle-tracking TTE, while feature-tracking using magnitude images acquired with 4D flow MRI shows good correlation with the previous technique, having the advantage of an integrated one-sequence approach comprising both flow and functional information [123].

Regarding aortic measurements in BAVD patients from childhood onward, which are of utmost importance in initial and follow-up evaluations of aortopathy, MRI can offset the drawbacks of TTE, as it is more accurate and reproducible, especially for the thoracic aorta distal to the root [122]. Morphologic evaluation and structural classification of the bicuspid aortic valve demonstrate strong agreement in aortopathy phenotype distribution, with small differences in ascending aorta and aortic root diameter measurements between MRI black-blood, white-blood, and TTE, but with clinical impact, supporting the need for constant, longitudinal multimodal imaging surveillance in patients with BAVD to ensure optimized management and decision-making [124]. When TTE employs the leading-edge-to-leading-edge convention, the measurements of aortic root and ascending aorta diameters align closely and consistently with the internal diameters obtained via CMR, showing mean differences of 0.4 ± 3.5 mm (*p* = 0.852) [125]. Conversely, TTE based on the inner-edge-to-inner-edge convention significantly underestimates all aortic diameters compared to CMR (*p* < 0.0001) [125]. In a large, population-based study, both CMR and TTE demonstrated high reproducibility for measurements of the sinus of Valsalva and sinotubular junction, although CMR showed slightly better performance, while agreement for the aortic annulus was only moderate (r = 0.51–0.57) [126]. The agreement between TTE and CMR varies notably depending on BAV morphotype and root geometry. TTE tends to underestimate the aortic root diameter more in right-noncoronary (RN) cusp fusion patterns than in right-left (RL) fusion, with mean differences of −2.8 ± 2.8 mm versus 0.1 ± 2.5 mm (*p* < 0.001) [127]. In raphe-type BAV, the mean absolute differences between TTE and CMR are significantly higher in asymmetric than in symmetric aortic roots (3.3 ± 2.2 mm vs. 1.6 ± 1.9 mm, *p* = 0.002) [127].

4D-flow CMR represents one of the most comprehensive noninvasive techniques currently available for the assessment of complex three-dimensional cardiovascular flow patterns, whereas conventional cardiac ultrasound provides only fundamental hemodynamic data depending on Doppler techniques, despite its advantages (easy, accessible, noninvasive technique) compared to higher costs, prolonged acquisition times, and the need for sedation or general anesthesia in CMR, which are extremely challenging, particularly in pediatric patients [104]. 4D flow MRI is a valuable research tool for analyzing abnormal aortic hemodynamics in pediatric BAV patients, but its clinical use is limited by small, heterogeneous studies and lack of long-term data. It helps identify hemodynamic biomarkers predicting aortopathy progression before anatomical changes occur. Studies show it detects elevated ascending aortic velocity and WSS even in the absence of valvulopathy, revealing stable patient-specific flow derangements suitable for serial monitoring [115,117,128]. A study of 53 patients found that abnormal velocity and WSS correlated with aortic Z-scores, whereas conventional metrics showed weak associations [119]. Patients with valvulopathy had higher velocity and WSS, linked to LV remodeling [117], whereas those with repaired coarctation showed that baseline velocity and WSS predicted aortic narrowing [129]. However, clinical adoption faces obstacles such as small, heterogeneous samples, inconsistent methods, and errors due to partial volume effects. No large pediatric trials have assessed long-term outcomes associated with these biomarkers. Currently, 4D flow MRI is primarily a research tool, slowly integrating into clinical surveillance, but not yet validated for risk stratification. Its ability to visualize flow, measure WSS, and evaluate valve function offers valuable insights into bicuspid aortopathy [130,131,132].

The main advanced and primarily research-oriented parameters evaluated through 4D-flow MRI are briefly explained in Table 4 [32,45,133]. Also, the main results from studies evaluating blood flow abnormalities by using CMR in pediatric BAV-aortopathy are synthesized in Table 3 [96,114,115,116,117,118,119].

Overall, key diagnostic values of cardiovascular magnetic resonance imaging in BAVD reside in the ability to ensure unparalleled aortic visualization, thickness assessment, mass measurements, 4D flow quantification and hemodynamic analysis and profiling, flow and pressure gradient assessment, WSS mapping, 3D morphological mapping, comprehensive assessment of valvular anatomy and structure, qualitative and quantitative evaluation of valvular lesions (regurgitation/stenosis), comprehensive functional and geometrical ventricular assessment (chamber quantification by evaluating wall thickness, mass, volumes), and myocardial tissue characterization by assessment of perfusion, viability, fibrosis, scar, remodeling, and deformation.

### 3.6. Pediatric Versus Adult Management of Bicuspid Aortic Valve Disease—Current Guidelines

Current available guidelines on aortopathy in pediatric subjects, and particularly in BAVD, are still limited, with one paper elaborated by the American Heart Association on cardiovascular management of aortopathy in children [6], and two available guidelines for daily clinical practice addressed to primary care providers [7], and cardiologists and cardiologic surgeons [8]. The American Association for Thoracic Surgery issued consensus guidelines on bicuspid aortic valve-related aortopathy, drawing primarily on adult studies [3]. Additionally, it has attracted interest from many other researchers, who have performed valuable work addressing this topic [91,134]. However, dealing with such a complex entity remains challenging, with utmost importance in the realm of pediatric cardiovascular care and significant concerns regarding the role of patient phenotype, the associated risks, and the most appropriate parameter to optimize treatment decisions [135].

One of the cornerstones of BAVD management is the structural and functional assessment of the aortic valve and aorta, as potential complications derive from valvular dysfunction or vascular enlargement and may tailor the surveillance strategy and the therapeutic decisions. As for aorta measurements, in adults, end-diastolic leading-edge to leading-edge is the method of choice for transthoracic echocardiography, whereas for CT/MRI, end-diastolic outer wall-to-outer wall measurements or inner wall-to-inner wall dimensions can be conducted, with various methods remaining under debate, such as sinus-to-sinus or Laplace methods for aortic root sizing in patients undergoing surveillance for thoracic aortic disease [3,136]. In pediatric patients, on the other hand, measurements are taken perpendicular to the aortic flow in the parasternal long axis imaging plane during systole, with the aortic valve leaflets open to their maximum dimension, using the inner-edge-to-inner-edge method in 2D mode, and then they are normalized using automatically calculated scores, such as Z score using traditional and new nomograms (Pediatric Heart Network, Halifax), newly introduced Q score based on a machine-learning algorithm [98,137].

For uncomplicated BAV with normal valve function, pediatric patients generally have clinical and echocardiographic check-ups every 2 years. Typically, a comprehensive 2D transthoracic echocardiogram provides a full assessment [108]. When BAV is linked with aortic dilation, cardiac MRI is increasingly used in pediatric cases to measure aortic sizes and flow issues [108]. Follow-up intervals for children increase with age, starting at about 7 months in newborns and reaching 28 months at age 18 for those with asymptomatic, isolated BAV without stenosis, regurgitation, or dilation [138]. Using adult guidelines as a model might result in more frequent follow-up and imaging than necessary for children, considering the low progression rate of the disease [58].

Regarding medical treatment and lifestyle management, over 80% of pediatric practitioners address aortic regurgitation or dilation medically, using beta-blockers or angiotensin II receptor blockers to potentially slow aortic growth, guided by data from Marfan syndrome studies [3,138]. Retrospective research indicates that treatment with losartan or atenolol can reduce the rate of aortic expansion compared to no treatment in children with BAV [32,139]. Most children and adolescents with uncomplicated BAV do not need restrictions on sports, and recreational physical activity is generally safe for affected children [108,140]. Guidelines highlight the importance of managing activity and exercise in pediatric patients, with exercise restrictions usually considered when echocardiographic mean gradients reach around 38 mmHg [32,138].

Concerning surgical thresholds and interventions, adult guidelines recommend aortic replacement as a Class I procedure when the aortic diameter reaches or exceeds 55 mm. However, flexible thresholds of 50 mm or even 45 mm may be considered based on individual patient factors, such as BAV morphology, severity of valve dysfunction, presence of coarctation, growth rate, and family history [91,141]. Both adult and pediatric patients follow similar guidelines for the aortic diameter at which surgery becomes necessary, although specific pediatric criteria are not well established [8]. Isolated dilation of the aortic root or ascending aorta rarely warrants surgery in children and adolescents, and such interventions are uncommon in this group [32,47]. The 2024 AHA scientific statement recommends customizing surgical thresholds for the root or ascending aorta in children based on genetic mutations and high-risk profiles [32]. In children and adolescents with BAV, aortic valve stenosis and/or regurgitation are the main reasons for intervention, rather than dilation alone [47]. Surgery is generally advised once stenosis or regurgitation becomes severe, regardless of age, but is typically not performed in younger children (birth to age 6) with severe dilation [138]. The Ross procedure, originally used mainly for pediatric patients, is increasingly employed in adults and remains a suitable option for children, adolescents, and select adults under 50 years old [2,8].

Most long-term BAV complications occur in adulthood, underscoring the need for a structured transition from pediatric to adult congenital cardiology, especially for young patients with complex BAV [108,142]. Pediatric care should consider somatic growth, the risk of re-intervention, and anticoagulation management tailored to each patient, in addition to the expertise of surgeons and institutions [8].

The main differences regarding the management of pediatric and adult patients with BAVD, based on the previously mentioned current guidelines, are summarised in Table 5.

## 4. Concluding Remarks

Building on these recent advances, this narrative review confirms that BAVD and related conditions are complex disorders driven by the intricate interplay between genetic predisposition, cellular and molecular mechanisms, and hemodynamic factors. This review delves deeper into the multifaceted clinical presentations and circulating biomarkers, combined with high-definition, noninvasive, functional multimodal imaging, which have great potential to enhance diagnostic accuracy, risk stratification, and cost-effectiveness of care in pediatric bicuspid aortopathy. However, further prospective studies on larger pediatric populations are warranted to establish their clinical utility compared with current standard evaluation.

Strengths: A major strength of this review is its comprehensive approach, which focuses on a relatively neglected area represented by pediatric patients with BAVD and integrates findings across different levels of research, thus providing significant clinical implications, especially in improving the management of BAV from childhood.

Limitations: This review has certain limitations that should be acknowledged. First, we deliberately opted for a narrative review format, as it provides the flexibility needed to discuss the interplay among various mechanisms, clinical presentations, and potential predictive factors for progression of BAVD-related complications, beginning in childhood. Consequently, this review is qualitative, so we did not perform a quantitative synthesis, such as meta-analyses. Selection of included studies was based on the authors’ knowledge, which could introduce bias, and some relevant studies may have been missed. Finally, as the literature in this field evolves rapidly, new evidence may have emerged.

Future Perspectives: Given the heterogeneity of the current literature, future systematic reviews could focus on more targeted topics, such as the potential role of specific circulating biomarkers and imaging tools in predicting future complications of BAVD in pediatric patients. Future clinical practices could involve national and international registries with these patients’ data, aiding better management. Future scientific inquiry should prioritize longitudinal studies that investigate the progression profiles of valvular and vascular disorders associated with BAV in children. In conclusion, although knowledge on this topic continues to grow, several critical areas remain insufficiently explored, particularly in children and young adults.

## Figures and Tables

**Table 1:** Key differences between BAV and TAV aortopathy.

**Feature**	**BAV Aortopathy**	**TAV Aortopathy**
Epidemiological aspects	- frequent in BAV population - onset earlier in life; more prevalent at younger ages, with aortopathy detectable even in newborns - reduced association with risk factors for aortopathy	- rare in general population - onset later in life (>70 years) - association with hypertension and other risk factors for aortopathy
Valve anatomy	- 2 cusps instead of 3 cusps - moderate correlation with severity of aortic stenosis	- 3 cusps - lower correlation with severity of aortic stenosis - lower prevalence of aortic stenosis
Aortic macroscopic features and dimensions	- larger ascending aortic diameters - associated with coarctation of the aorta - associated with type of cusp fusion	- commonly symmetric dilation of the tubular ascending aorta
Microscopic characteristics and histopathology of the aorta	- distinct aortic wall histological features resembling Marfan syndrome - normal fiber architecture - medial degeneration and lower fibrillin content - loss of smooth muscle cells with apoptosis, phenotype switching and MMP secretion	- severe elastin degeneration - cystic degeneration of the media - inflammatory response often present
Biomechanical properties	- similar impaired mechanical properties and aortic wall fragility	
Blood flow disturbances	- eccentric, helicoidal and turbulent flow pattern - high-velocity outflow jets - elevated regional WSS	- central and symmetric flow - high-velocity eccentric jets - symmetrical, low and stable WSS
- Clinical implications:		
Risk of adverse vascular events	- higher aortic dissection risk - comparable or lower rates of adverse aortic events - higher prevalence of aortic root aneurysm, dilation of the outer curve of the ascending aorta - Aortopathy and dissection occasionally occur after AVR.	- aortopathy and dissection rarely occur after AVR
Management	- more common aortic surgery, with a standard surgical threshold of ≥55 mm, but intervention is often considered at ≥50 mm (or ≥45 mm) if risk factors are present, such as a family history of aortic dissection, rapid growth (>5 mm/year), or concomitant severe valve dysfunction - younger age undergoing AVR - more favorable prognosis after AVR or aortic root replacement, but with higher late reoperation rates	- surgical threshold for aortic dimension is ≥ 55 mm for asymptomatic patients without concomitant connective tissue disorders or rapid aortic enlargement pattern

Abbreviations: BAV, bicuspid aortic valve; TAV, tricuspid aortic valve; WSS, wall shear stress; MMP, matrix metalloproteinase; AVR, aortic valve replacement.

**Table 2:** Summary of the main genes and epigenetic factors involved in the pathogenesis of BAV-associated aortopathy.

**Etiopathogenetic Factor**	**Gene/MicroRNA**	**Main Roles**
Gene	*GATA 4, 5, 6* *NOTCH1* *EGFR,* *TGFBR2*	embryological formation of BAV
	*BGN* *COL1A1, 2* *COL3A1* *EFEMP2* *ELN* *FBN1* *ROBO4*	extracellular matrix remodeling
	*ACTA2* *MYH11* *FOXE3* *MAT2A* *MYLK* *PRKG1*	vascular smooth cell apoptosis and switching phenotype from contractile to secretory
	*FBN1* *NOTCH1* *SK1* *SLC2A10* *SMAD2, 3, 4, 6* *TGFB2, 3* *TGFBR 1, 2*	dysregulation of TGF-β signaling pathways
Epigenetic factors	miR-1, miR-21, miR-122, miR-130a, miR-486	malformation of the aortic valve
	miR-718, miR-17, miR-106a, miR-15b, miR-20a	regulation of aorta dimensions
	miR-34a	integrity of the vascular wall
	miR-195-5p	aortic valve calcification
	*NOTCH1* hypermethylation	abnormal valve cell osteogenesis and smooth muscle remodeling

**Table 4:** Definitions of the main advanced parameters used in the evaluation of BAV using 4D-flow MRI.

**Parameter**	**Definition**	**Reference**
WSS (wall shear stress)	The force (N) per unit area (m^2^) exerted by a moving fluid in the direction of the local tangent of the tubular surface, particularly the force that blood flow exerts on the vessel wall as a function of viscosity and vessel geometry.	[45]
OSI (oscillatory shear index)	The quantification of the change in direction and magnitude of WSS.	[45]
SFD (systolic flow displacement)	The distance between the vessel centreline node and the forward velocity-weighted center of mass position. It measures the displacement of the blood flow from the centerline of the aorta, indicating flow eccentricity.	[45]
sFRR (systolic flow reversal ratio/ retrograde flow)	The quantification of the degree of retrograde flow, or backward blood flow during systole.	[133]
EL (viscous energy loss)	The energy dissipated within blood flow due to frictional forces.	[32]
KE (kinetic energy)	The quantification of flow velocity and energy dynamics within the aorta.	[32]

**Table 5:** Key claims comparing pediatric and adult BAV-associated aortopathy.

**Feature**	**Pediatric Patients**	**Adult Population**
Age at presentation	- 18 years for early-onset complications	- onset before 70 years old
Predominant valvular dysfunction	- aortic regurgitation predominates	- aortic stenosis is more frequent
Aortic measurement	- greater variability in aortic dimensions - absolute size and various scores used for normalisation of retrieved values according to body mass index, body surface area	- relatively constant rate of growth - absolute size measured by imaging tools
Aortic growth rate	- increases with age and peaks around 8–9 years in isolated BAV	- progresses steadily with age
Hemodynamic disturbance	- WSS and velocity direction correlate with ascending aorta Z-scores	- WSS magnitude is associated with dilation of the aorta
Evolution and progression (rate of complications)	- lower aortic dissection risk	- higher risk of acute aortic disease and valvular dysfunction
Family screening	- recommended in all first-degree relatives of patients with BAV, using echocardiography	
Follow-up through cardiovascular imaging intervals, based on dilatation of the aortic root or ascending aorta	- Regular visits every 1 to 2 years for mild and stable cases: - Z score < 2—every 12–24 months - Z score = 2–3.5—every 12–18 months - More frequent checks every 6 to 12 months for moderate or active valvular or vascular aortic dysfunction: - Z score 4–4.5—every 6–12 mo - Z score > 4.5—every 6–12 mo	- yearly reassessment of the entire thoracic aorta if greater than 45 mm - measurement of the thoracic aorta segments at 6 months, especially if other risk factors are present (aortic coarctation or family history of dissection).
Genetic testing indications	- personal history of aortic or arterial dissection/rupture or spontaneous bowel perforation - clinical features suggesting heritable thoracic aortic disease (HTAD) first-degree relative with a pathogenic or likely pathogenic variant in an HTAD gene - family member with history of aortic or arterial dissection/rupture, aortic aneurysm or bowel perforation - Ascending aortic Z score ≥ 5 - Aortic root Z score ≥ 3.5 - Aortic root or ascending aorta ≥ 4 cm - Aortic root phenotype (aortic root Z score > ascending aorta Z score) - family history of BAV, HLHS	
Sports restriction	- Higher risk: - moderate to severe valvular disease - avoid contact sports (e.g., tackle football, hockey, wrestling) and high-intensity training and competitions - avoid strenuous training and prolonged physical exertion, heavy weightlifting, and sustained exertion to exhaustion - avoid intense training or competition for highly select teams	- individualized exercise prescription or restrictions - avoidance of strenuous - lifting, pushing, or straining in cases of previously repaired aortic dissection, as well as strenuous strength training - avoidance of weight lifting, competitive athletics involving isometric exercise in patients with moderately dilated aortas (>45 mm) or rapid growth rate of aorta dimensions - Aerobic or endurance exercise is recommended in patients with aortopathy. - no restrictions in the absence of valvular dysfunction or aortic dilatation
Medical therapy	- beta-blockers used for slowing aortic dimension growth in selected cases *	- antihypertensive therapy for blood pressure control - beta-blockers or calcium-channel blockers in patients with chronic aortic dissection
Percutaneous interventions	- balloon valvuloplasty is recommended for severe aortic stenosis in selected cases	- percutaneous balloon aortic valvuloplasty is rarely used in severe calcific aortic stenosis, mainly as a temporary bridge to definitive transcatheter or surgical aortic valve
Surgical procedures	- absolute dimensions of the aorta are primarily used to guide the timing of surgical intervention, with a surgical threshold of 5–5.5 cm, depending on the presence of risk factors #	- repair of the ascending aorta/root is recommended when the aortic diameter is ≥55 mm in patients without risk factors and ≥50 mm in patients with risk factors
Literature data	- limited resources, with most results extrapolating from studies and guidelines on the adult population	- larger cohort studies and specific guidelines on the management of BAV and associated disorders and complications

Abbreviations: BAV, bicuspid aortic valve; RN WSS, wall shear stress; HTAD, heritable thoracic aortic disease; HLHS, hypoplastic left heart syndrome. * Limited data; medical treatment for aortic *Z* score ≥ 4 is reasonable. # Additional risk factors: rapid growth > 3 mm/year, predominant aortic root dilation (root phenotype), coarctation, family history of dissection.

## Data Availability

No new data were created or analyzed in this study. Data sharing is not applicable.

## Abbreviations

The following abbreviations are used in this manuscript:

2D	two-dimensional
4D-flow MRI	four-dimensional flow magnetic resonance imaging
ADMA	asymmetric dimethylarginine
AP	antero-posterior cusp fusion phenotype
AR	aortic valve regurgitation
AS	aortic valve stenosis
AVR	aortic valve replacement
BAVD	bicuspid aortic valve disease
BMP	bone morphogenic pathway
CD	color Doppler
CMR	cardiovascular magnetic resonance
CT	computed tomography
CTA	computed tomography angiography
CW	continuous wave Doppler
FGF	fibroblast growth factor
LL	latero-lateral cusp fusion phenotype
LN	left-non coronary cusp fusion phenotype
LVEDV	left ventricular end-diastolic volume
LVESV	left ventricular end-systolic volume
MLR	monocyte-to-lymphocyte ratio
MMP	matrix metalloproteinase
MRI	magnetic resonance imaging
PIV	pan-immune-inflammation value
PW	blood tissue pulse wave Doppler
RL	right-left cusp fusion phenotype
RN	right-non-coronary cusp fusion phenotype
SIRI	Systemic Inflammatory Response Index
sRAGE	soluble receptor for advanced glycation end-products
TAV	tricuspid aortic valve
TAVI	transcatheter aortic valve implantation
TGF-β	transforming growth factor-β
TIMP	tissue inhibitor of metalloproteinase
TTE	transthoracic echocardiography
Wnt	wingless-related MMTV integration sites
WSS	wall shear stress
